# Parameter analysis using swallowing sounds shows differences in bolus volume, bolus viscosity, sex, and age

**DOI:** 10.1038/s41598-025-13877-5

**Published:** 2025-08-20

**Authors:** Yuka Konoike, Kei Naramura, Takahiro Hasegawa, Izumi Tsukayama, Saya Maruoka, Takayo Kawakami, Kayoko Ishii, Tomoya Yoshida, Masaki Hokari, Toshiko Suzuki-Yamamoto

**Affiliations:** 1 Department of Nutritional Science, Okayama Prefectural University, 111 Kuboki, Soja, 719-1197 Okayama Japan; 2 Department of Nutrition and Health Science, Fukuyama University, 985-1 Sanzo, Higashimura-cho, Fukuyama, 729-0292 Hiroshima Japan; 3 Department of Human Information Engineering, Okayama Prefectural University, 111 Kuboki, Soja, 719-1197 Okayama Japan

**Keywords:** Swallowing sounds, Cervical auscultation, Acoustic analysis, Measurement, Deglutition, Deglutition disorders, Geriatrics, Nutrition, Quality of life

## Abstract

Acoustic analysis of pharyngeal sounds during swallowing may capture physiological functions, providing a noninvasive method for early screening of swallowing decline. In this study, we examined changes in swallowing function across variations in bolus volume, bolus viscosity, sex, and age using parameters such as swallowing duration, average voltage, and swallowing power derived from swallowing sounds. The results showed that average voltage and swallowing power were significantly higher in men, both in younger (aged 20–25 years) and older (aged 50–65 years) groups, regardless of bolus volume and viscosity. These parameters generally decreased as bolus viscosity increased, with significant differences observed in all participants except older men. Regarding swallowing duration, older men took significantly longer to swallow a high-viscosity bolus compared to younger men, whereas no age-related differences were observed in women. This finding suggests that men experience greater age-related deterioration in swallowing than women. Overall, this simple and noninvasive measurement method appears to be an effective and objective tool for evaluating swallowing function. It is capable of detecting alterations associated with sex and aging, as well as changes in swallowing status related to the physical properties of dysphagia diets.

**Supplementary Information:**

The online version contains supplementary material available at 10.1038/s41598-025-13877-5.

## Introduction

Early and accurate diagnosis of swallowing status is essential to prevent aspiration and ensure adequate food intake, thereby helping avoid malnutrition and frailty in older adults. While gold-standard instrumental assessments, such as fiberoptic endoscopic evaluation of swallowing (FEES) and videofluorographic swallowing study (VFSS)^1,2^ provide detailed diagnostic information, these modalities require qualified personnel at medical institutions, and their invasive nature poses certain risks^3^. In contrast, simple evaluation methods based on physical assessments such as water swallowing test and repeated saliva swallowing test can be easily performed at the clinical bedside or in-home care settings^4–6^. However, these methods make objective and quantitative evaluation of swallowing function and food suitability challenging. Recently, alternative methods, such as cervical auscultation and acoustic analysis, have shown promise for assessing swallowing function^7–12^. Swallowing acoustic analysis–based parameters may serve as useful and objective screening tools for dysphagia assessment.

Although the use of cervical auscultation as a stand-alone tool is controversial because of assertion that it could be rated based on the human ear alone, high-resolution cervical auscultation (HRCA) based on recording of swallowing vibrations and sounds using accelerometers and microphones, has shown promising results in categorizing normal and disordered swallowing independent of human input^13,14^. Additionally, Donohue et al. reported that HRCA is effective as a monitoring system for future dysphagia treatment^15^. If swallowing function could be measured through HRCA and/or acoustic analysis, it can serve to enhance the screening process and can lead to earlier diagnosis of dysphagia. In the future, simpler, noninvasive, and quantitative methods for measuring swallowing function will be essential. Therefore, quantifying the acoustic characteristics of swallowing through parameter analysis is important for developing an objective measurement tool.

Few studies have quantified the relationship between swallowing sounds and food properties in alternative methods. Previously, we demonstrated that a parameter derived from swallowing sounds—specifically, the power of the swallowing sound signal, calculated as the time integral of voltage—was inversely correlated with food hardness. This correlation was observed for sol-like foods, which are colloidal solutions that retain their liquid properties and do not require chewing, as assessed by texture profile analysis^16^. These findings indicate that swallowing sound parameters can reflect differences in food properties during swallowing. However, sex and age differences were not analyzed in the previous study.

In this study, we aimed to develop a parameter analysis derived from swallowing sounds and to establish a simple, noninvasive, and objective method for measuring physiological swallowing function. We analyzed changes in swallowing parameters based on differences in bolus volume, bolus viscosity, sex, and age. In this study, we used a condenser microphone with settings optimized for capturing swallowing sounds, analyzing the recordings with equations developed in MATrix LABoratory (MATLAB) (The MathWorks, Inc., Natick, MA) rather than relying on human senses. This study aligns with HRCA in capturing pharyngeal sounds during swallowing as a biological signal, which we believe can be used as an adjunct in evaluating swallowing function. This proposed method will be useful not only for measuring individual swallowing function but also for identifying foods tailored to the swallowing abilities of both older and younger individuals.

## Methods

### Participants

Before the study commenced, the methods were reviewed and approved by the Ethics Committee of Okayama Prefectural University. We confirm that all methods and procedures were performed in accordance with relevant guidelines and regulations. We recruited two groups of different ages (20’s and over 50 years) from students and faculty members at Okayama Prefectural University. After obtaining informed consent from all participants, they were screened using a health questionnaire^17^. Thirty-two healthy adults participated in the study, with no reported issues specific to chewing or swallowing functions. They were categorized into two age groups.

### Instrumentation

**Fig. 1:**
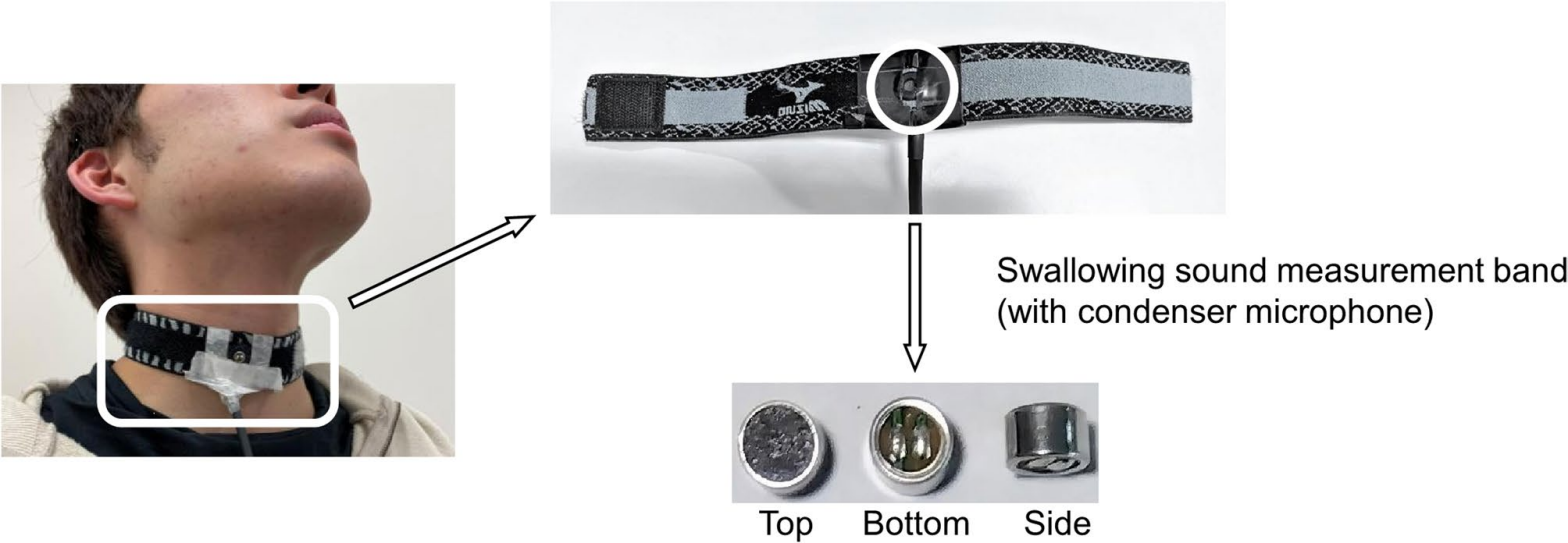
Signal measured by the microphone during swallowing sound collection.

To measure swallowing sounds, a condenser microphone (XCM-6035, SPL LIMITED, Kowloon, Hong Kong) was used (Fig. 1). Owing to its small size (6.0 · 3.4 mm), it does not interfere with participants’ swallowing. Additionally, its frequency response range of 20 Hz–16 kHz makes it suitable for capturing swallowing sounds within the frequency range of 30 Hz–1 kHz^18^. Anticipating variations in the magnitude of participants’ swallowing sounds, the acoustic signals were amplified using an inverting amplifier circuit with a gain of 50. The amplified signal was then converted to digital format through AD conversion at a sampling frequency of 20 kHz and recorded using a data logger (GL2000, Graphtec Co., Ltd., Yokohama, Japan). Analysis was conducted using MATLAB.

### Procedures

The experiment was conducted in a quiet, room-temperature environment with minimal background noise. Participants were seated upright in a straight-backed chair throughout the swallowing experiment. Following the method developed by Takahashi et al.^19^ the condenser microphone was placed over the lateral border of the trachea, just below the cricoid cartilage, and secured with an elastic band (12JY6S03, Mizuno Corp., Osaka, Japan) (Fig. 1, informed consent was obtained to publish the image in an online open access publication). Participants placed each bolus in their mouth and swallowed naturally at the experimenter’s signal. Three trials were conducted for each bolus type. The following boluses were provided. For the bolus volume experiment, varying amounts of mineral water (3, 5, 10, and 15 mL) were served in 3/4 oz cups, based on previous studies that experimented with varying bolus volumes to examine swallowing properties^7,20–22^. For the bolus viscosity experiment, thickened liquids of different viscosities were prepared using 5 mL of mineral water and 1.0% and 3.5% TROMELIN V (NUTRI Co., Ltd., Yokkaichi, Japan). Table 1 shows the National Dysphagia Diet (NDD) standard^23^ and International Dysphagia Diet Standardization Initiative (IDDSI)^24^ levels used to corroborate the thickness of the prepared boluses. Table 2 shows the viscosities and syringe flow test values of 1.0% and 3.5% TROMELIN V. The viscosities of 1.0% and 3.5% TROMELIN V were 125.3–163.2 mPa·s (141.12 ± 9.44, nectar-like, IDDSI Level 2 Mildly Thick) and 729–850 mPa·s (793.11 ± 27.90, honey-like, IDDSI Level 3 Moderately thick), respectively, based on five separate measurements for each experiment (mean ± standard deviation (SD)). The syringe flow test values of 1.0% and 3.5% TROMELIN V were 7.83 ± 0.05 mL (IDDSI Level 2, Mildly thick) and < 10.0 ± 0 (IDDSI Level 3, Moderately thick), respectively, based on three separate measurements for each experiment (mean ± SD).

**Table 1:** National dysphagia diet standard^23^ and international dysphagia diet standardization initiative (IDDSI)^24^.

NDD^23^		IDDSI^24^	
Standardized levels	Viscosity (cP)	Thickness level	Syringe flow test (mL)
Thin liquid	1–50	0 (Thin)	<1.0
		1 (Slightly thick)	1.0–4.0
Nectar-like	51–350	2 (Mildly thick)	4.0–8.0
Honey-like	351–1750	3(Moderately thick)	>8.0
Spoon-thick	>1750	4(Extremely thick)	10.0

**Table 2:** Viscosity and syringe flow test value of thickened liquids used in the experiments.

Concentration (%)	Viscosity (mPa s)	Syringe flow test (mL)
1.0%	125.3–163.2 (141.12 ± 9.44)	7.83 ± 0.05
3.5%	729–850 (793.11 ± 27.90)	< 10.0 ± 0 *

* 10 mL on the scale, but decreased from 10 mL in 10 s, so below 10 mL was recorded as “<10.0”. Data are presented as mean ± SD.

The swallowing sound signal for each trial was recorded immediately before the experimenter’s cue and continued for 10 s. The recording was saved to a data logger for later analysis.

### Viscosity analysis

Prior to the swallowing experiment, the viscosity of the 1.0% and 3.5% thickened liquids used in the bolus viscosity experiment was measured using a viscometer (VISCOMETER TV-25; Toki Sangyo, Co., Ltd., Tokyo, Japan) featuring a cone diameter of 28 mm and an angle of 3°. Viscosity measurements were taken after 1 min under the following conditions: temperature of 20 °C and shear rate of 50 s^− 1^, following the method established by the Japanese Society of Dysphagia Rehabilitation Dysphagia Diet Committee 2021^25^. Each measurement was performed five times.

### Syringe flow test

The IDDSI^24^ flow test was used to measure the flow properties of 1.0% and 3.5% thickened liquids in this study. A 10 mL Tip syringe (TERUMO Co., Ltd., Tokyo, Japan) was used instead of the preferred syringe model (BD 303134, Becton Dickinson Medical Pte., Ltd., Singapore) because there are few practical problems with measurement error depending on the type of syringe within a difference of less than ± 10% from the measured viscosity value^26^. During the measurement process, the syringe nozzle was pointed down and covered with a finger while the syringe was precisely filled with the test sample up to the 10 mL mark. The finger was then removed from the nozzle for precisely 10 s before closing the nozzle again to measure the remaining sample volume. Each measurement was performed five times.

### Acoustic analysis

To evaluate swallowing parameters from the microphone-measured signals, three features were used: (1) swallowing duration, (2) voltage proportional to swallowing sound pressure, and (3) swallowing power, defined as the time integral of the voltage. Raw data from the microphone contained ripple components of AC power and biological noise such as body movements and pulse. These were removed using a digital filter. The analysis time was set to 3 s, including 1.5 s before and after the maximum voltage occurred.

To extract the peak values of sound pressure and time intervals between each peak, the signal was smoothed using a simple moving average. The swallowing sound signal was assumed to consist of *n* discrete values ${y_i}~\left( {i=1,~2,~3, \cdots ,n} \right)$. Because the amplitude ${y_i}$ could take positive or negative values, the voltage ${v_i}~\left( {i=1,~2,~3, \cdots ,n} \right)$ proportional to swallowing sound pressure was defined by the following equation:

1 $${v_i}=\left| {{y_i}} \right|$$

Using the simple moving average, Eq. (1) was smoothed by setting *m* = 1500 in Eq. (2):

2 $$Vo{s_i}=\frac{1}{{m+1}} \cdot \mathop \sum \limits_{{j= - \frac{m}{2}}}^{{\frac{m}{2}}} {v_{i+j}}$$

As the result of Eq. (2) contained an offset from the moving average, the offset was removed using Eq. (3):

3 $${V_i}=Vo{s_i} - \frac{1}{{l+1}}~ \cdot \mathop \sum \limits_{{j=k}}^{{k+l}} Vo{s_{i+j}}$$

Here, the offset was defined as the average voltage from 8 to 10 s after starting measurement of the digitally filtered signal. In Eq. (3), the data at 8 s corresponds to the 160000th data point (*k* = 160,000), and the data at 10 s corresponds to the 200000th data point (*l* = 40,000). Therefore, the microphone measurements were converted into a voltage signal proportional to swallowing sound pressure using Eqs. (1) to (3).

Finally, three features were extracted to evaluate swallowing. Using Eq. (4), the area ${S_i}$ of the swallowing sound signal in a given micro interval was calculated from ${V_i}$ and the sampling interval ${t_s}=5 \cdot {10^{ - 6}}~{\text{s}}.$

4 $${S_i}={t_s} \cdot {V_i}$$

For a 3 s analysis time, the total area *S* of the swallowing sound was obtained as follows:

$$S=\mathop \sum \limits_{{i=1}}^{N} {S_i}$$

The value increased with swallowing. For a curve $S~\left( t \right)$, the following process allowed us to obtain the rate of change in the curve’s slope at a given time *t*. Here, ${t_1}$ was defined as the time when the rate of change in the slope first equaled or exceeded 0.4, and ${t_2}$ was defined as the time when it finally equaled or decreased below − 0.4. The threshold (± 0.4) was determined experimentally by (P1) approximating differentiation using differences, (P2) smoothing using moving averages (*m* = 2000), and (P3) calculating the numerical gradient using differences.

Therefore, the swallowing duration was determined as the interval from $\:{t}_{1}$​ to $\:{t}_{2}$. Furthermore, the average voltage proportional to swallowing sound pressure during swallowing duration as well as the swallowing power, calculated as the time integral of the voltage during the swallowing duration, were obtained.

### Statistics

Data were statistically evaluated using analysis of variance with the Bonferroni or Dunnett test, applying significance levels of *p* < 0.05 or *p* < 0.01. All statistical analyses were performed using KaleidaGraphWin 4.5 (Synergy Software, Reading, PA, USA).

## Results

### Parameter analysis using swallowing sounds

**Fig. 2:**
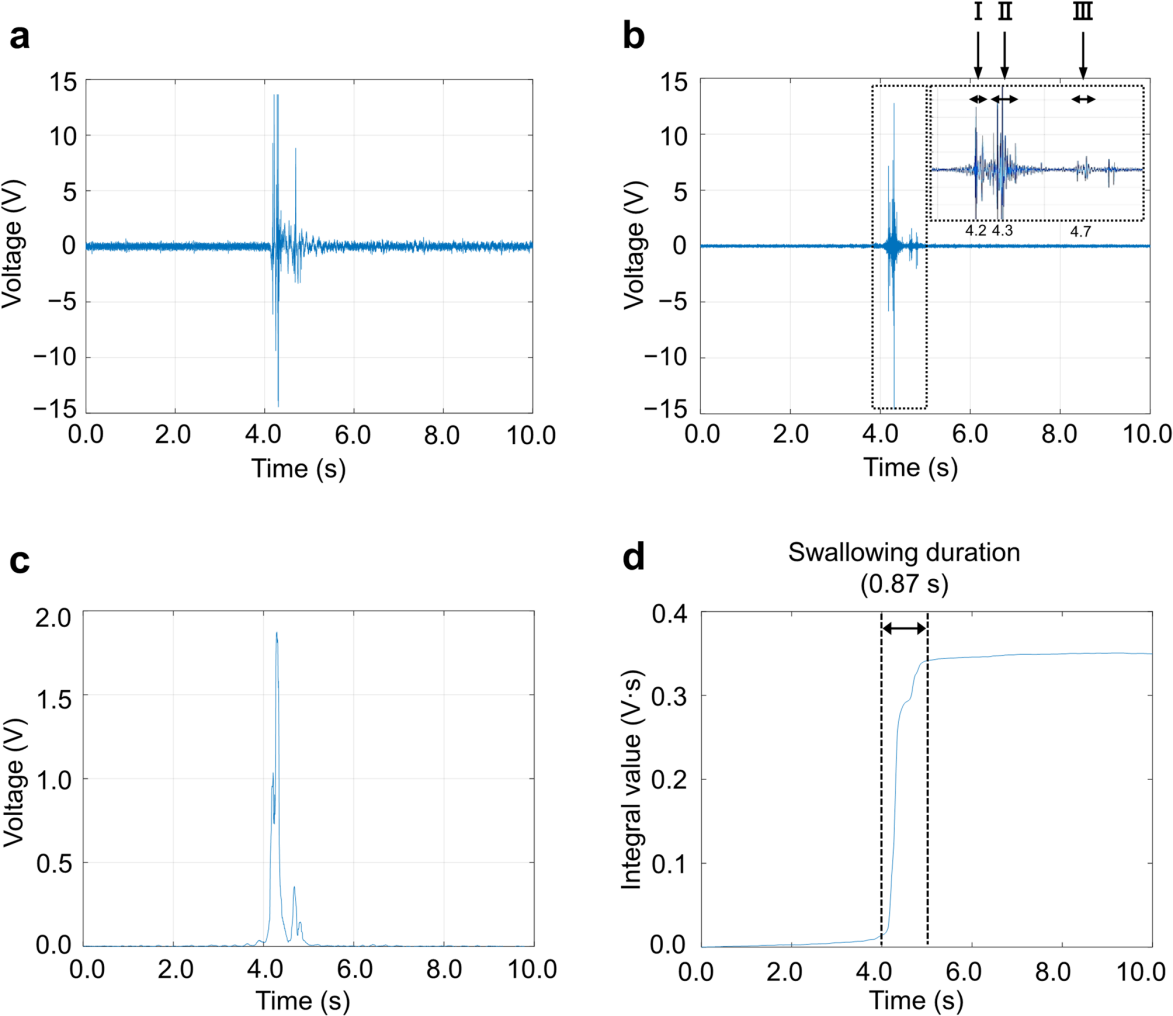
A typical signal waveform of swallowing sound and parameter transformation trace. The original signal (a) from the microphone contained noise and was processed with a digital filter to obtain a cleaner signal (b). The waveform within the dashed line in (b) is enlarged in the inset, showing that the swallowing sound consists of three components: I, II, and III. Since signal (b) corresponds to sound pressure in voltage, it was transformed by taking the absolute value to obtain signal (c). From signal (c), the swallowing power (d), calculated as the time integral of the voltage, was derived. Furthermore, signal (d) was differentiated and smoothed using a moving average, and the numerical gradient was calculated from differences, allowing for the determination of the swallowing start and end times.

Swallowing sounds were measured as participants drank water, and the typical acoustic analysis results are shown in Fig. 2a–d. The analysis of this typical acoustic signal using MATLAB indicates that the raw signal (a) from the microphone shows amplitude increases at approximately 4 s from the start of measurement, with a decrease at around 5 s. The signal peaks appear three times at approximately 4.2, 4.3, and 4.7 s (b). Signal (c) shows the voltage corresponding to sound pressure, and (d) illustrates the temporal variation of swallowing power. Swallowing power increased concurrently with the occurrence of swallowing sounds, with no change during times without swallowing. Based on this, three features related to swallowing sound were determined: (1) Swallowing duration, calculated using methods (P1) to (P3) as described in the “Acoustic Analysis” section of the Methods, was 0.87 s. (2) The average voltage, proportional to the swallowing sound pressure within the swallowing duration, was 0.41 V. (3) Swallowing power, the time integral of the voltage within the swallowing duration, was 0.32 V·s. This measurement system and parameter analysis were used in this study’s experiments.

### Effect of bolus volume on swallowing parameters

In the younger group (aged 20–25 years), which included eight men (mean ± SD, 22.25 ± 1.09 years) and eight women (mean ± SD, 22.25 ± 1.20 years), individual normal swallowing sounds were recorded, and changes in swallowing sound parameters—namely, swallowing duration, average voltage, and swallowing power—were analyzed across different bolus volumes.

To investigate the effect of bolus volume, water was used as the test material, and the analysis was limited to the younger group due to safety and ethical considerations. Although participants of the older group reported no swallowing difficulties, a previous study has shown that the incidence of silent aspiration increases with larger bolus volumes even in healthy older adults^27^. Therefore, the bolus volume manipulation was not performed in the older group to minimize potential health risks.

**Fig. 3:**
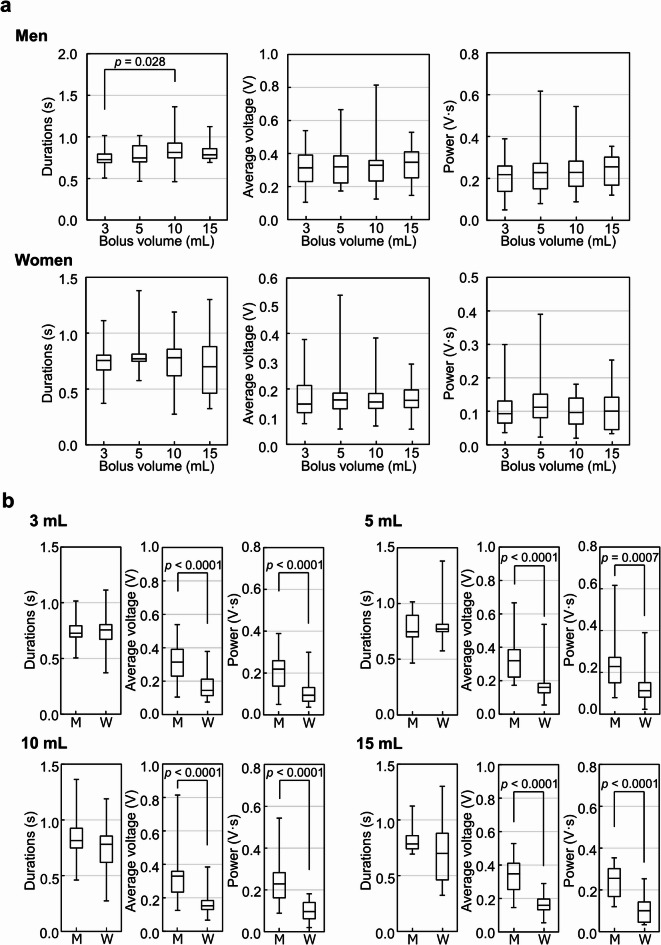
Changes in swallowing sound parameters in the younger group (aged 20–25 years). Effect of bolus volume on swallowing sound parameters (a), and comparison of swallowing sound parameters between men and women (b). Swallowing duration (s), average voltage (V), and swallowing power (V·s) were analyzed for each bolus volume (3, 5, 10, and 15 mL of water) were analyzed as described in the Methods. The plots represent the median (horizontal line) ± interquartile range (IQR) from eight participants (men or women), each with three measurements (*n* = 24). The Bonferroni or Dunnett test was used. M: men, W: women.

No significant changes in swallowing parameters were found among the bolus volumes for each man and woman, except that swallowing duration tended to increase with larger bolus volumes in men, showing a significant difference between the 3 and 10 mL boluses (*p* = 0.028) (Fig. 3a). Individual variability in swallowing duration was found to be greater in women than in men.

The average voltage and swallowing power remained stable across bolus volumes in both men and women. The individual mean ± SD across three trials for each bolus are shown in Supplementary Table S1 and 2 online.

In comparing swallowing parameters between men and women, the average voltage and swallowing power were significantly higher in men than in women for all bolus volumes (5 mL swallowing power was *p* = 0.0007, others *p* < 0.0001); however, no differences in swallowing duration were found (Fig. 3b). At bolus volumes of 3, 5, 10, and 15 mL, the average voltage was 2.16-, 1.99-, 2.16-, and 2.19-fold higher and swallowing power was 2.34-, 2.03-, 2.37-, and 2.54-fold higher in men than in women, respectively.

### Effect of bolus viscosity on swallowing parameters

The younger group (aged 20–25 years) included eight men (mean ± SD: 22.25 ± 1.09 years) and eight women (mean ± SD: 22.25 ± 1.20 years). The older group (aged 50–65 years) included eight men (mean ± SD: 58.00 ± 3.84 years) and eight women (mean ± SD: 54.13 ± 3.10 years). There was no significant difference in age between sexes (*p* = 0.057).

**Fig. 4:**
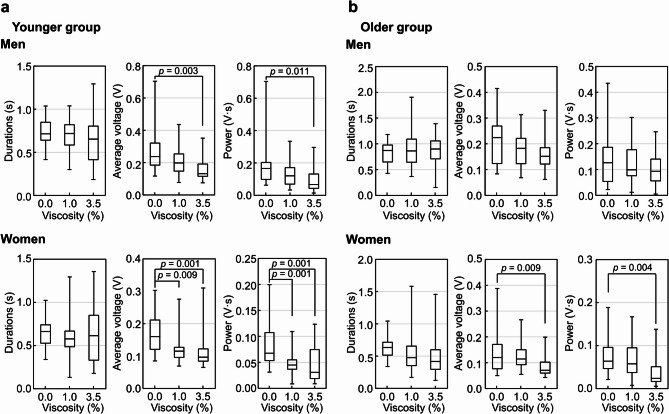
Effect of bolus viscosity on swallowing sound parameters in the younger (aged 20–25 years) and older (aged 50–65 years) groups, separated by sex. Given the observed sex differences in the bolus volume experiment (Fig. 3), the analysis was stratified by age group and sex to clarify the differential effects of viscosity on swallowing physiology. In both younger and older groups, women showed greater changes in average voltage and swallowing power in response to variations in bolus viscosity compared to men, suggesting sex-related differences in physiological responses. Swallowing duration (s), average voltage (V), and swallowing power (V·s) were analyzed in the younger (a) and older (b) groups by sex. Boluses with 0.0%, 1.0%, and 3.5% concentrations of the thickening agent TROMELIN V were swallowed, with viscosities as shown in Table 2. Data are from thirty-two participants, eight per group by age and sex. The plots represent the median (horizontal line) ± IQR from three measurements (*n* = 24). 0.0%: water, 1.0%: IDDSI Level 2 Mildly Thick, 3.5%: IDDSI Level 3 Moderately thick.

In the younger group, increasing bolus viscosity resulted in a decrease in both average voltage and swallowing power (Fig. 4a). For men, the 3.5% (IDDSI Level 3 Moderately thick) bolus showed a significant decrease in average voltage (44.54%, *p* = 0.003) and swallowing power (60.04%, *p* = 0.011) compared to the 0.0% bolus. In women, the 1.0% (IDDSI Level 2 Mildly Thick) and 3.5% boluses demonstrated significant decreases in average voltage, with reductions of 27.9% (*p* = 0.009) and 39.7% (*p* = 0.001), respectively, and in swallowing power, with reductions of 33.7% (*p* = 0.001) and 54.3% (*p* = 0.001), respectively, compared to the 0.0% bolus. Conversely, the effect of bolus viscosity on swallowing parameters was weaker in the older group than in the younger group (Fig. 4b). In older women, all swallowing parameters decreased as bolus viscosity increased; in particular, the average voltage and swallowing power for the 3.5% bolus showed significant decreases of 41.3% (*p* = 0.009) and 62.3% (*p* = 0.004), respectively, compared to the 0.0% bolus. For older men, both average voltage and swallowing power tended to decrease with increasing bolus viscosity; however, no significant differences were observed among the swallowing parameters. In both younger and older groups, women’s average voltage and swallowing power were more susceptible to changes in bolus viscosity than those of men.

**Fig. 5:**
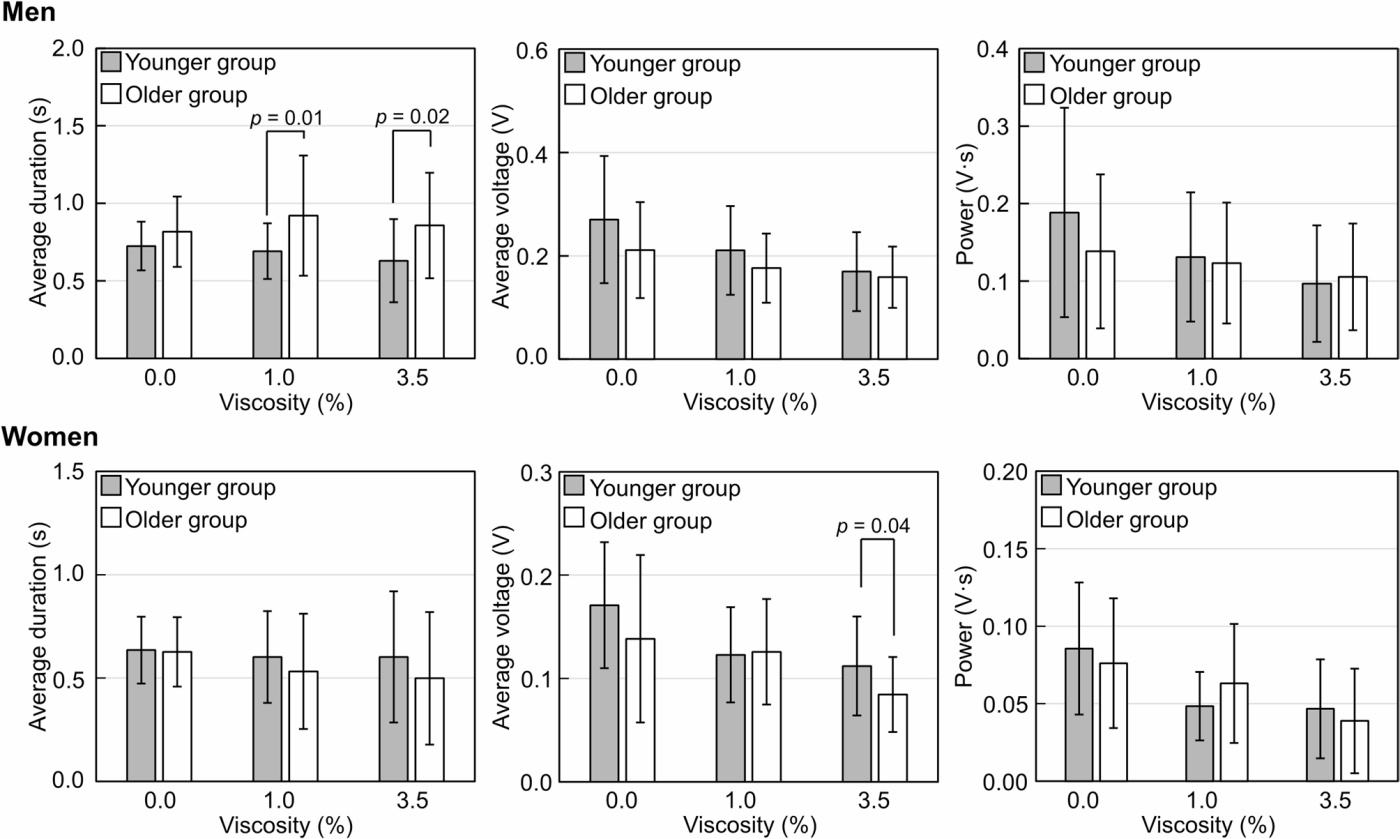
Comparison of swallowing sound parameters between the younger (aged 20–25 years) and older (aged 50–65 years) groups at each bolus viscosity, separated by sex. Given the observed sex differences in the bolus volume experiment (Fig. 3), we present the differential effects of viscosity on swallowing physiology stratified by age group and sex. It is indicated that the effect of aging on swallowing function differs between men and women. Average swallowing duration (s), average voltage (V), and swallowing power (V·s) were analyzed at each bolus viscosity (0.0%, 1.0%, and 3.5% concentrations of the thickening agent TROMELIN V), with corresponding viscosities shown in Table 2. Data are from thirty-two participants, eight per group by age and sex. Data are presented as the mean ± SD from three measurements (*n* = 24). 0.0%: water, 1.0%: IDDSI Level 2 Mildly Thick, 3.5%: IDDSI Level 3 Moderately thick.

Figure 5 shows a comparison of swallowing parameters by sex in the younger and older groups provided with boluses of differing viscosities. Among men, the average voltage and swallowing power for all boluses with different viscosities did not differ significantly between the younger and older groups. However, average swallowing duration significantly increased with higher bolus viscosity only in the older group, which also had a significantly longer swallowing duration than the younger group for the 1.0% bolus (1.3-fold, *p* = 0.01) and the 3.5% bolus (1.4-fold, *p* = 0.02). Conversely, among women, most swallowing parameters showed no difference between age groups, except for the average voltage at the 3.5% bolus, where the older group showed a 24.6% lower average voltage compared to the younger group (*p* = 0.04). These results indicate that the effect of aging on swallowing function differs between men and women, as reflected in the changes in swallowing parameters. Specifically, aging may extend swallowing duration in men, whereas women may respond less noticeably to this change, showing little difference across generations.

**Fig. 6:**
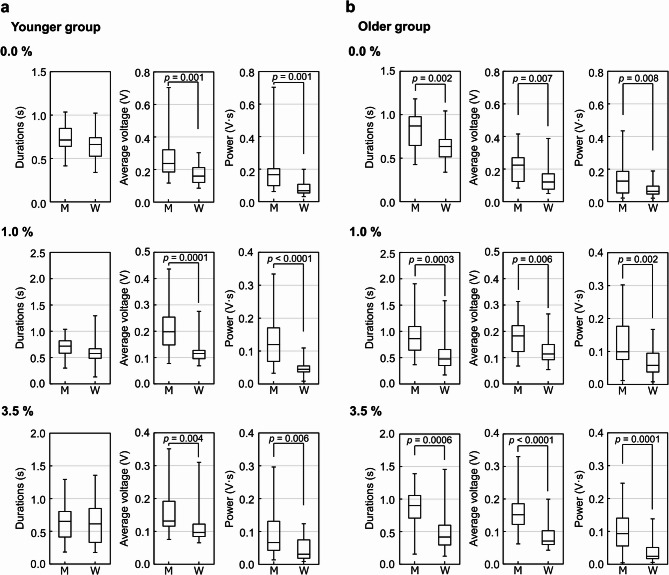
Comparison of swallowing sound parameters between men and women at each bolus viscosity. Swallowing duration (s), average voltage (V), and swallowing power (V·s) were analyzed in the younger (aged 20–25 years) (a) and older (aged 50–65 years) (b) groups. Boluses with 0.0%, 1.0%, and 3.5% concentrations of the thickening agent TROMELIN V were swallowed, with viscosities as shown in Table 2. Data are from thirty-two participants, eight per group by age and sex. The plots represent the median (horizontal line) ± IQR from three measurements (*n* = 24). M: men, W: women. 0.0%: water, 1.0%: IDDSI Level 2 Mildly Thick, 3.5%: IDDSI Level 3 Moderately thick.

Figure 6 shows a comparison of swallowing parameters between men and women for each bolus concentration. In the younger group, no significant difference was observed in swallowing duration between men and women, however, average voltage and swallowing power were significantly higher in men than in women (*p* < 0.01). In the older group, all parameters—swallowing duration, average voltage, and swallowing power—were significantly higher in men than in women (*p* < 0.01).

In the younger group, the increases in each parameter when men swallowed 0.0%, 1.0%, and 3.5% boluses compared to women were as follows: average voltage increased by 1.48-fold (*p* = 0.001), 1.72-fold (*p* = 0.0001), and 1.36-fold (*p* = 0.004); and swallowing power increased by 2.46-fold (*p* = 0.001), 2.67-fold (*p* < 0.0001), and 2.15-fold (*p* = 0.006), respectively (Fig. 6a).

In the older group, the increases in each parameter when men swallowed 0.0%, 1.0%, and 3.5% boluses compared to women were as follows: swallowing duration increased by 1.37-fold (*p* = 0.002), 1.81-fold (*p* = 0.0003), and 2.16-fold (*p* = 0.0006); average voltage increased by 1.86-fold (*p* = 0.007), 1.60-fold (*p* = 0.006), and 2.15-fold (*p* < 0.0001); and swallowing power increased by 1.97-fold (*p* = 0.008), 1.71-fold (*p* = 0.002), and 3.86-fold (*p* = 0.0001), respectively (Fig. 6b).

Thus, a similar tendency was found in both younger and older groups, with average voltage and swallowing power being higher in men than in women. Although swallowing duration was similar in younger men and women, this was not observed in the older group, where men had markedly longer swallowing durations. These results suggest that men are more susceptible to the effects of aging on swallowing function, particularly in terms of swallowing duration.

The individual mean ± SD across three trials for each bolus are shown in Supplementary Table S3–6 online.

## Discussion

In this study, we aimed to establish a simple, noninvasive, and objective method for measuring physiological swallowing function by capturing swallowing sounds as a biological signal and introducing novel parameters that reflect the key acoustic characteristics of normal swallowing physiology. We analyzed changes in swallowing parameters based on differences in bolus volume, bolus viscosity, sex, and age. The proposed measurement method converted swallowing sounds into parameters such as swallowing duration, average voltage, and swallowing power to evaluate swallowing functions, demonstrating its accuracy in measuring differences in bolus viscosity. Additionally, this method effectively highlighted differences in swallowing function across sex and age groups.

Previous studies have reported that swallowing sounds consist of three distinct components corresponding to specific physiological events during swallowing^10,16,28–31^. Our data (Fig. 2b) exhibited a similar three-phase acoustic pattern. The first sound likely corresponds to the elevation of the larynx and hyoid bone as the bolus moves into the pharynx. The second sound appears to reflect the entry of the bolus into the hypopharynx and opening of the upper esophageal sphincter. The third sound may indicate the return of the hyoid and larynx to their resting positions and the closure of the sphincter. Although we did not use concurrent instrumental methods such as videofluoroscopy or endoscopy, the acoustic patterns observed in our study closely resemble those described in previous literature. Therefore, we suggest that the recorded sounds are consistent with known physiological events associated with swallowing.

Some studies have reported a relationship between bolus volume and swallowing duration. Cichero et al. found that swallowing duration decreased as bolus volume increased^7^whereas Honda et al.^10^ and Youmans et al.^20^ reported that swallowing duration increased with larger bolus volumes. Our findings similarly show a slight increase in swallowing duration in men, with a significant difference between the 3 and 10 mL boluses. In women, however, there was a high degree of individual variation, and no correlation was observed between bolus volume and swallowing duration.

In this study, bolus volume was varied only in the younger group, whereas no volume escalation was attempted in the older group. We refrained from varying bolus size in the older group because a previous study has shown that larger volumes increase the risk of silent aspiration even in ostensibly healthy older adults. A previous study has demonstrated that silent aspiration is not uncommon in healthy older adults and that the risk increases with larger bolus volumes^27^. As our methodology did not include real-time imaging (e.g., VFSS or endoscopy), we adopted a conservative approach to ensure participant safety and limited the bolus volume to 5 mL in the older group. The results from the younger group demonstrated that a 5 mL bolus provided swallowing sound recordings with a sufficient signal-to-noise ratio and adequate parameter sensitivity for our analytic system. Accordingly, all subsequent experiments, including those involving the older group, employed a fixed 5 mL volume to maintain measurement sensitivity while minimizing potential risk.

Furthermore, some studies reported no difference in swallowing duration between men and women^7,20,32,33^ whereas others indicated that women have shorter swallowing durations than men^19,34^ leaving findings on sex differences inconclusive. However, these studies on sex differences in swallowing duration have not been compared across age groups^7,19,20,32–34^. Our results showed that swallowing durations were significantly longer in older men than in older women, but no differences were observed between younger men and women (Fig. 6). These findings suggest a potential interaction between age and sex, and the results of this study may indicate that sex differences in swallowing duration vary among age groups and contribute to understanding the acoustic characteristics of normal swallowing physiology.

Additionally, Youmans et al. reported that increased viscosity led to longer swallowing durations^20^ which contrasts with our results, where swallowing duration remained stable regardless of bolus viscosity in both men and women. This difference may result from methodological variations, as our study used a condenser microphone to capture biosignals during swallowing, whereas theirs employed an accelerometer. When comparing age groups in our study (Fig. 5), swallowing duration in men tended to be longer in the older group than in the younger group, particularly with higher bolus viscosities. Conversely, swallowing duration in women showed no change despite age differences. Previous studies have shown that swallowing duration increases with age^7,20,22,32,35,36^possibly due to age-related neuromuscular decline, including lower laryngeal position, sarcopenia, and general slowing of central nervous system function^35–37^. Dantas et al. demonstrated that behavioral changes in swallowing with age are more pronounced in men than in women^38^. Our findings suggest that men may be more susceptible to the effects of aging on swallowing duration.

In the present study, the average voltage and swallowing power were also analyzed and showed no changes with bolus volume in both men and women, indicating that bolus volume does not affect these parameters. Cichero et al. similarly found that swallowing sound intensity remained stable and was unaffected by bolus volume^7^. Although the specific parameters differed, our results support their findings, showing that average voltage and swallowing power, both related to swallowing intensity, were not influenced by bolus volume. The average voltage and swallowing power were significantly higher in men than in women across all bolus volumes (Fig. 3b). Previous studies have suggested that the maximum intensity of the acoustic signal, a parameter similar to swallowing power, showed no difference between men and women^7^ although other findings indicated that intensity may vary by sample^20^. Differences in parameter definitions could contribute to these variations. Compared with the maximum intensity of the acoustic signal, swallowing power may better represent the force applied during swallowing and thus more readily reflect the burden imposed by the bolus on the participant.

In the experiment examining factors influencing swallowing parameters by bolus viscosity (Fig. 4), the average voltage and swallowing power tended to decrease as bolus viscosity increased, with significant differences between 0.0% and 3.5% observed in the younger group and older women. However, older men did not show significant differences with increasing viscosity, though a decreasing trend was noted. This may indicate that older men have reduced sensitivity in swallowing function in response to changes in bolus viscosity. Additionally, the lack of significant changes in average voltage and swallowing power across bolus viscosities in older men may reflect compensatory muscular activity. It is possible that stronger contractions were required to swallow more viscous boluses, generating acoustic signals comparable to those with thinner consistencies. This interpretation is supported by the longer swallowing durations observed in older men, suggesting increased effort to maintain bolus transit. However, the observed changes in average voltage and swallowing power may reflect alterations in the resonant properties of the pharyngeal cavity as boluses of varying viscosities pass through. Therefore, the lower values of these parameters with thicker boluses may not necessarily indicate a decrease in swallowing muscle power. Because electromyographic data were not collected concurrently in this study, we cannot definitively attribute these changes to variations in muscular effort. Accordingly, interpretations related to muscle power during swallowing should be approached with caution. Future studies incorporating electromyography or imaging techniques may provide further insights into the physiological basis of these acoustic parameters.

Previously, it was demonstrated that the power of the swallowing sound signal inversely correlated with the hardness of food that does not require chewing^16^. The present results align with this finding, showing that swallowing power reflects bolus viscosity. It is possible that more power is required to maintain a cohesive bolus as it becomes more prone to breaking up with increased viscosity. Youmans et al. reported that the greatest intensity of the acoustic signal, a parameter related to swallowing, was louder when swallowing thin liquids compared to thicker ones^20^. Our parameter, swallowing power, is likely similar to the parameter they described.

By comparing each swallowing sound parameter by bolus viscosity (Fig. 6), we found that the average voltage and swallowing power were significantly higher in men than in women in both the younger and older groups across all bolus viscosities. Younger men showed higher parameters than younger women for each bolus volume. These results indicate that average voltage and swallowing power were consistently higher in men than in women, regardless of bolus volume and viscosity.

In this study, a novel measurement system and parameters were proposed, demonstrating the potential to physiologically capture changes in swallowing due to variations in food properties. In the future, this method could serve as a screening tool for identifying individuals at risk of declining swallowing function and could aid in producing dysphagia diets tailored to individual needs. However, this study was limited by the number and age range of participants, and further studies with a broader and larger participant group are necessary. Additionally, the food properties measured using this method were compared only among a few thickening agents of different viscosities; therefore, further evaluation using foods with a wider range of properties is needed.

## Conclusions

This study examined the acoustic characteristics of normal swallowing physiology. In the proposed method for assessing physiological swallowing function, swallowing sounds are collected as biological signals and converted into parameters for evaluating swallowing function. We believe this method can accurately detect differences in bolus viscosity. An increase in bolus viscosity resulted in significant decreases in average voltage and swallowing power in all participants, except for older men. Furthermore, this method clarified differences related to sex and age. Our results indicate sex differences in swallowing parameters. In the younger group, men showed significantly higher average voltage and swallowing power than women, but no difference in swallowing duration. In contrast, in the older group, men exhibited significantly higher values than women for all parameters. In comparisons by age, swallowing duration was significantly longer in older men compared to younger men, whereas no significant difference was observed between older and younger women. These findings suggest that men may be more susceptible to age-related changes in swallowing function. This simple, noninvasive measurement method can serve as useful and objective screening evaluation tool. It may be applied as a screening method to help avoid risks such as aspiration in individuals with potential functional decline and to provide tailored foods according to each individual’s swallowing function.

## Supplementary Information

Below is the link to the electronic supplementary material.

Supplementary Material 1

## Data Availability

The datasets analysed during the current study are available from the corresponding author on reasonable request.

## Abbreviations

FEES: Fiberoptic endoscopic evaluation of swallowing
HRCA: High-resolution cervical auscultation
IDDSI: International Dysphagia Diet Standardization Initiative
IQR: Interquartile range
MATLAB: MATrix LABoratory
SD: Standard deviation
VFSS: Videofluorographic swallowing study
