# Supplementary material for: Parameter analysis using swallowing sounds shows differences in bolus volume, bolus viscosity, sex, and age
Source: Sci Rep. 2025 Aug 20;15:30639. doi: 10.1038/s41598-025-13877-5 (PMC12368031; doi:10.1038/s41598-025-13877-5)
Supplement: Supplementary file 1 — Supplementary Material 1 [file 41598_2025_13877_MOESM1_ESM.docx]

**Supplementary Information**

**Parameter analysis using swallowing sounds shows differences in bolus volume, bolus viscosity, sex, and age**

Yuka Konoike^1,2^, Kei Naramura^3^, Takahiro Hasegawa^3^, Izumi Tsukayama^1^, Saya Maruoka^1^, Takayo Kawakami^1^, Kayoko Ishii^2^, Tomoya Yoshida^3^, Masaki Hokari^3*^, and Toshiko Suzuki-Yamamoto^1*^

^1^Department of Nutritional Science, Okayama Prefectural University, 111 Kuboki, Soja, Okayama, 719-1197, Japan

^2^Department of Nutrition and Health Science, Fukuyama University, 985-1 Sanzo, Higashimura-cho, Fukuyama, Hiroshima, 729-0292, Japan

^3^Department of Human Information Engineering, Okayama Prefectural University, 111 Kuboki, Soja, Okayama, 719-1197, Japan

* **Correspondence:** Masaki Hokari and Toshiko Suzuki-Yamamoto

E-mail: mhokari@ss.oka-pu.ac.jp, toshiko@fhw.oka-pu.ac.jp

**Supplementary Table S1** Swallowing sound parameters according to bolus volume in the younger group (aged 20–25 years men)

| Parameters | Participant | Bolus volume | | | | | | | |
| --- | --- | --- | --- | --- | --- | --- | --- | --- | --- |
|  | No. | 3 mL | | 5 mL | | 10 mL | | 15 mL | |
|  |  | Mean | SD | Mean | SD | Mean | SD | Mean | SD |
| Swallowing duration (s) | M1 | 0.73 | 0.08 | 0.89 | 0.02 | 1.04 | 0.21 | 1.05 | 0.04 |
|  | M2 | 0.79 | 0.05 | 0.72 | 0.02 | 0.81 | 0.11 | 0.82 | 0.05 |
|  | M3 | 0.67 | 0.12 | 0.51 | 0.06 | 1.02 | 0.24 | 0.78 | 0.05 |
|  | M4 | 0.86 | 0.11 | 0.92 | 0.04 | 0.85 | 0.11 | 0.71 | 0.01 |
|  | M5 | 0.69 | 0.02 | 0.71 | 0.02 | 0.72 | 0.02 | 0.77 | 0.04 |
|  | M6 | 0.63 | 0.05 | 0.67 | 0.10 | 0.68 | 0.15 | 0.75 | 0.02 |
|  | M7 | 0.72 | 0.04 | 0.78 | 0.09 | 0.89 | 0.06 | 0.79 | 0.03 |
|  | M8 | 0.79 | 0.04 | 0.93 | 0.06 | 0.83 | 0.05 | 0.95 | 0.13 |
|  | Total | 0.74 | 0.06 | 0.77 | 0.05 | 0.86 | 0.12 | 0.83 | 0.05 |
| Average voltage (V) | M1 | 0.27 | 0.04 | 0.21 | 0.00 | 0.19 | 0.02 | 0.18 | 0.03 |
|  | M2 | 0.28 | 0.02 | 0.36 | 0.03 | 0.27 | 0.03 | 0.34 | 0.05 |
|  | M3 | 0.17 | 0.05 | 0.24 | 0.02 | 0.22 | 0.08 | 0.24 | 0.01 |
|  | M4 | 0.48 | 0.06 | 0.54 | 0.12 | 0.55 | 0.19 | 0.43 | 0.08 |
|  | M5 | 0.40 | 0.03 | 0.36 | 0.04 | 0.37 | 0.02 | 0.43 | 0.01 |
|  | M6 | 0.21 | 0.02 | 0.20 | 0.02 | 0.24 | 0.02 | 0.31 | 0.05 |
|  | M7 | 0.38 | 0.04 | 0.40 | 0.03 | 0.44 | 0.07 | 0.38 | 0.04 |
|  | M8 | 0.32 | 0.00 | 0.32 | 0.06 | 0.35 | 0.01 | 0.35 | 0.06 |
|  | Total | 0.31 | 0.03 | 0.33 | 0.04 | 0.33 | 0.06 | 0.33 | 0.04 |
| Swallowing power (V·s) | M1 | 0.17 | 0.02 | 0.15 | 0.00 | 0.16 | 0.04 | 0.15 | 0.02 |
|  | M2 | 0.19 | 0.02 | 0.23 | 0.02 | 0.19 | 0.02 | 0.25 | 0.05 |
|  | M3 | 0.09 | 0.03 | 0.10 | 0.01 | 0.17 | 0.05 | 0.17 | 0.00 |
|  | M4 | 0.38 | 0.01 | 0.47 | 0.13 | 0.42 | 0.09 | 0.28 | 0.06 |
|  | M5 | 0.26 | 0.02 | 0.23 | 0.03 | 0.24 | 0.02 | 0.31 | 0.02 |
|  | M6 | 0.12 | 0.01 | 0.12 | 0.03 | 0.15 | 0.04 | 0.21 | 0.04 |
|  | M7 | 0.25 | 0.02 | 0.29 | 0.02 | 0.37 | 0.08 | 0.28 | 0.04 |
|  | M8 | 0.24 | 0.02 | 0.27 | 0.04 | 0.27 | 0.02 | 0.30 | 0.02 |
|  | Total | 0.21 | 0.02 | 0.23 | 0.04 | 0.25 | 0.05 | 0.24 | 0.03 |

Data represents mean in three separate measurements

SD: standard deviation

| Parameters | Participant | Bolus volume | | | | | | | |
| --- | --- | --- | --- | --- | --- | --- | --- | --- | --- |
|  | No. | 3 mL | | 5 mL | | 10 mL | | 15 mL | |
|  |  | Mean | SD | Mean | SD | Mean | SD | Mean | SD |
| Swallowing duration (s) | W1 | 0.79 | 0.04 | 0.65 | 0.07 | 0.74 | 0.06 | 0.46 | 0.15 |
|  | W2 | 0.77 | 0.06 | 0.75 | 0.04 | 0.91 | 0.04 | 0.73 | 0.14 |
|  | W3 | 0.64 | 0.19 | 0.79 | 0.01 | 0.68 | 0.16 | 0.59 | 0.19 |
|  | W4 | 0.55 | 0.02 | 0.71 | 0.06 | 0.62 | 0.22 | 0.53 | 0.19 |
|  | W5 | 0.71 | 0.03 | 0.77 | 0.01 | 0.69 | 0.17 | 0.54 | 0.16 |
|  | W6 | 0.72 | 0.05 | 0.85 | 0.05 | 0.81 | 0.04 | 0.97 | 0.03 |
|  | W7 | 1.00 | 0.12 | 1.28 | 0.08 | 1.16 | 0.02 | 1.24 | 0.05 |
|  | W8 | 0.74 | 0.08 | 0.78 | 0.06 | 0.41 | 0.10 | 0.63 | 0.08 |
|  | Total | 0.74 | 0.07 | 0.82 | 0.05 | 0.75 | 0.10 | 0.71 | 0.12 |
| Average voltage (V) | W1 | 0.22 | 0.11 | 0.17 | 0.01 | 0.10 | 0.01 | 0.12 | 0.02 |
|  | W2 | 0.27 | 0.05 | 0.26 | 0.07 | 0.15 | 0.02 | 0.16 | 0.01 |
|  | W3 | 0.14 | 0.04 | 0.12 | 0.01 | 0.23 | 0.11 | 0.18 | 0.04 |
|  | W4 | 0.10 | 0.01 | 0.07 | 0.01 | 0.08 | 0.01 | 0.11 | 0.03 |
|  | W5 | 0.14 | 0.02 | 0.16 | 0.02 | 0.20 | 0.01 | 0.13 | 0.06 |
|  | W6 | 0.18 | 0.05 | 0.18 | 0.01 | 0.19 | 0.01 | 0.24 | 0.03 |
|  | W7 | 0.10 | 0.02 | 0.14 | 0.01 | 0.16 | 0.01 | 0.17 | 0.04 |
|  | W8 | 0.21 | 0.02 | 0.34 | 0.15 | 0.16 | 0.01 | 0.20 | 0.06 |
|  | Total | 0.17 | 0.04 | 0.18 | 0.04 | 0.16 | 0.02 | 0.16 | 0.04 |
| Swallowing power (V·s) | W1 | 0.16 | 0.10 | 0.09 | 0.0.1 | 0.06 | 0.01 | 0.04 | 0.00 |
|  | W2 | 0.18 | 0.04 | 0.17 | 0.06 | 0.11 | 0.02 | 0.10 | 0.02 |
|  | W3 | 0.07 | 0.01 | 0.07 | 0.01 | 0.12 | 0.03 | 0.09 | 0.01 |
|  | W4 | 0.04 | 0.00 | 0.03 | 0.01 | 0.03 | 0.01 | 0.04 | 0.01 |
|  | W5 | 0.09 | 0.02 | 0.10 | 0.01 | 0.12 | 0.03 | 0.09 | 0.04 |
|  | W6 | 0.11 | 0.04 | 0.13 | 0.01 | 0.14 | 0.03 | 0.21 | 0.03 |
|  | W7 | 0.08 | 0.02 | 0.15 | 0.01 | 0.16 | 0.01 | 0.19 | 0.04 |
|  | W8 | 0.13 | 0.01 | 0.24 | 0.11 | 0.06 | 0.01 | 0.11 | 0.02 |
|  | Total | 0.11 | 0.03 | 0.12 | 0.03 | 0.10 | 0.02 | 0.11 | 0.02 |

**Supplementary Table S2** Swallowing sound parameters according to bolus volume in the younger group (aged 20–25 years women)

Data represents mean in three separate measurements

SD: standard deviation

**Supplementary Table S3** Swallowing sound parameters according to bolus viscosity in the younger group (aged 20–25 years men)

| Parameters | Participant | Bolus viscosity | | | | | |
| --- | --- | --- | --- | --- | --- | --- | --- |
|  | No. | 0.0% | | 1.0% | | 3.5% | |
|  |  | Mean | SD | Mean | SD | Mean | SD |
| Swallowing duration (s) | M1 | 0.70 | 0.09 | 0.77 | 0.18 | 0.57 | 0.16 |
|  | M2 | 0.87 | 0.13 | 0.67 | 0.02 | 0.69 | 0.06 |
|  | M3 | 0.66 | 0.10 | 0.80 | 0.01 | 0.94 | 0.35 |
|  | M4 | 0.91 | 0.07 | 0.89 | 0.11 | 0.81 | 0.11 |
|  | M5 | 0.61 | 0.12 | 0.70 | 0.02 | 0.42 | 0.21 |
|  | M6 | 0.52 | 0.14 | 0.46 | 0.02 | 0.40 | 0.04 |
|  | M7 | 0.69 | 0.01 | 0.42 | 0.13 | 0.36 | 0.16 |
|  | M8 | 0.85 | 0.03 | 0.82 | 0.02 | 0.85 | 0.03 |
|  | Total | 0.73 | 0.09 | 0.69 | 0.06 | 0.63 | 0.14 |
| Average voltage (V) | M1 | 0.31 | 0.08 | 0.20 | 0.07 | 0.12 | 0.00 |
|  | M2 | 0.23 | 0.03 | 0.18 | 0.02 | 0.17 | 0.01 |
|  | M3 | 0.21 | 0.06 | 0.17 | 0.04 | 0.14 | 0.02 |
|  | M4 | 0.40 | 0.01 | 0.40 | 0.03 | 0.32 | 0.02 |
|  | M5 | 0.25 | 0.08 | 0.18 | 0.08 | 0.11 | 0.03 |
|  | M6 | 0.16 | 0.02 | 0.16 | 0.02 | 0.12 | 0.01 |
|  | M7 | 0.35 | 0.26 | 0.15 | 0.04 | 0.12 | 0.01 |
|  | M8 | 0.25 | 0.01 | 0.25 | 0.01 | 0.26 | 0.04 |
|  | Total | 0.27 | 0.07 | 0.21 | 0.04 | 0.17 | 0.02 |
| Swallowing power (V·s) | M1 | 0.21 | 0.08 | 0.13 | 0.05 | 0.06 | 0.01 |
|  | M2 | 0.17 | 0.02 | 0.09 | 0.02 | 0.09 | 0.02 |
|  | M3 | 0.11 | 0.04 | 0.12 | 0.03 | 0.09 | 0.03 |
|  | M4 | 0.33 | 0.03 | 0.32 | 0.02 | 0.23 | 0.05 |
|  | M5 | 0.13 | 0.06 | 0.10 | 0.05 | 0.03 | 0.02 |
|  | M6 | 0.07 | 0.01 | 0.06 | 0.01 | 0.04 | 0.0.1 |
|  | M7 | 0.30 | 0.29 | 0.05 | 0.01 | 0.03 | 0.02 |
|  | M8 | 0.18 | 0.01 | 0.18 | 0.01 | 0.20 | 0.04 |
|  | Total | 0.19 | 0.07 | 0.13 | 0.03 | 0.10 | 0.03 |

Data represents mean in three separate measurements

SD: standard deviation

**Supplementary Table S4** Swallowing sound parameters according to bolus viscosity in the younger group (aged 20–25 years women)

| Parameters | Participant | Bolus viscosity | | | | | |
| --- | --- | --- | --- | --- | --- | --- | --- |
|  | No. | 0.0% | | 1.0% | | 3.5% | |
|  |  | Mean | SD | Mean | SD | Mean | SD |
| Swallowing duration (s) | W1 | 0.61 | 0.06 | 0.42 | 0.21 | 0.58 | 0.08 |
|  | W2 | 0.67 | 0.07 | 0.65 | 0.11 | 0.27 | 0.05 |
|  | W3 | 0.37 | 0.04 | 0.82 | 0.16 | 0.58 | 0.16 |
|  | W4 | 0.48 | 0.13 | 0.46 | 0.07 | 0.97 | 0.29 |
|  | W5 | 0.65 | 0.10 | 0.55 | 0.06 | 0.41 | 0.18 |
|  | W6 | 0.76 | 0.05 | 0.60 | 0.05 | 0.72 | 0.19 |
|  | W7 | 0.88 | 0.11 | 0.69 | 0.43 | 1.04 | 0.16 |
|  | W8 | 0.66 | 0.02 | 0.63 | 0.14 | 0.24 | 0.04 |
|  | Total | 0.64 | 0.07 | 0.60 | 0.15 | 0.60 | 0.14 |
| Average voltage (V) | W1 | 0.10 | 0.01 | 0.12 | 0.01 | 0.10 | 0.03 |
|  | W2 | 0.13 | 0.02 | 0.10 | 0.02 | 0.09 | 0.00 |
|  | W3 | 0.23 | 0.04 | 0.08 | 0.01 | 0.10 | 0.04 |
|  | W4 | 0.16 | 0.03 | 0.14 | 0.02 | 0.10 | 0.01 |
|  | W5 | 0.13 | 0.03 | 0.11 | 0.01 | 0.08 | 0.01 |
|  | W6 | 0.26 | 0.03 | 0.10 | 0.02 | 0.18 | 0.09 |
|  | W7 | 0.16 | 0.04 | 0.21 | 0.07 | 0.11 | 0.01 |
|  | W8 | 0.19 | 0.06 | 0.11 | 0.01 | 0.12 | 0.03 |
|  | Total | 0.17 | 0.03 | 0.12 | 0.02 | 0.11 | 0.03 |
| Swallowing power (V·s) | W1 | 0.04 | 0.00 | 0.04 | 0.02 | 0.04 | 0.02 |
|  | W2 | 0.07 | 0.02 | 0.04 | 0.02 | 0.01 | 0.00 |
|  | W3 | 0.08 | 0.02 | 0.04 | 0.01 | 0.03 | 0.01 |
|  | W4 | 0.06 | 0.01 | 0.06 | 0.00 | 0.08 | 0.01 |
|  | W5 | 0.06 | 0.03 | 0.04 | 0.00 | 0.02 | 0.01 |
|  | W6 | 0.17 | 0.02 | 0.03 | 0.01 | 0.08 | 0.03 |
|  | W7 | 0.10 | 0.03 | 0.09 | 0.02 | 0.08 | 0.00 |
|  | W8 | 0.10 | 0.03 | 0.04 | 0.01 | 0.02 | 0.01 |
|  | Total | 0.09 | 0.02 | 0.05 | 0.01 | 0.05 | 0.01 |

Data represents mean in three separate measurements

SD: standard deviation

**Supplementary Table S5** Swallowing sound parameters according to bolus viscosity in the older group (aged 50–65 years men)

| Parameters | Participant | Bolus viscosity | | | | | |
| --- | --- | --- | --- | --- | --- | --- | --- |
|  | No. | 0.0% | | 1.0% | | 3.5% | |
|  |  | Mean | SD | Mean | SD | Mean | SD |
| Swallowing duration (s) | M9 | 0.88 | 0.01 | 0.84 | 0.02 | 1.19 | 0.16 |
|  | M10 | 0.94 | 0.17 | 1.64 | 0.22 | 0.90 | 0.40 |
|  | M11 | 0.80 | 0.17 | 0.67 | 0.04 | 0.77 | 0.09 |
|  | M12 | 0.54 | 0.06 | 1.24 | 0.26 | 0.94 | 0.12 |
|  | M13 | 0.95 | 0.20 | 0.89 | 0.02 | 0.96 | 0.15 |
|  | M14 | 0.89 | 0.05 | 0.46 | 0.07 | 0.24 | 0.11 |
|  | M15 | 0.47 | 0.05 | 0.54 | 0.08 | 0.72 | 0.21 |
|  | M16 | 1.07 | 0.06 | 1.09 | 0.02 | 1.15 | 0.13 |
|  | Total | 0.82 | 0.10 | 0.92 | 0.09 | 0.86 | 0.17 |
| Average voltage (V) | M9 | 0.32 | 0.04 | 0.23 | 0.01 | 0.21 | 0.02 |
|  | M10 | 0.26 | 0.04 | 0.16 | 0.05 | 0.20 | 0.09 |
|  | M11 | 0.25 | 0.04 | 0.18 | 0.01 | 0.18 | 0.02 |
|  | M12 | 0.14 | 0.04 | 0.14 | 0.02 | 0.14 | 0.03 |
|  | M13 | 0.12 | 0.01 | 0.13 | 0.01 | 0.18 | 0.05 |
|  | M14 | 0.09 | 0.01 | 0.07 | 0.00 | 0.07 | 0.01 |
|  | M15 | 0.22 | 0.07 | 0.25 | 0.04 | 0.12 | 0.02 |
|  | M16 | 0.29 | 0.09 | 0.24 | 0.04 | 0.18 | 0.02 |
|  | Total | 0.21 | 0.04 | 0.18 | 0.02 | 0.16 | 0.03 |
| Swallowing power (V·s) | M9 | 0.24 | 0.04 | 0.16 | 0.02 | 0.19 | 0.05 |
|  | M10 | 0.18 | 0.01 | 0.17 | 0.10 | 0.10 | 0.01 |
|  | M11 | 0.16 | 0.03 | 0.09 | 0.01 | 0.10 | 0.01 |
|  | M12 | 0.05 | 0.02 | 0.11 | 0.02 | 0.08 | 0.03 |
|  | M13 | 0.07 | 0.01 | 0.07 | 0.00 | 0.13 | 0.07 |
|  | M14 | 0.04 | 0.01 | 0.02 | 0.00 | 0.01 | 0.00 |
|  | M15 | 0.09 | 0.04 | 0.12 | 0.04 | 0.05 | 0.01 |
|  | M16 | 0.30 | 0.10 | 0.25 | 0.04 | 0.19 | 0.01 |
|  | Total | 0.14 | 0.03 | 0.12 | 0.03 | 0.11 | 0.02 |

Data represents mean in three separate measurements

SD: standard deviation

**Supplementary Table S6** Swallowing sound parameters according to bolus viscosity in the older group (aged 50–65 years women)

| Parameters | Participant | Bolus viscosity | | | | | |
| --- | --- | --- | --- | --- | --- | --- | --- |
|  | No. | 0.0% | | 1.0% | | 3.5% | |
|  |  | Mean | SD | Mean | SD | Mean | SD |
| Swallowing duration (s) | W9 | 0.74 | 0.22 | 0.97 | 0.43 | 0.89 | 0.40 |
|  | W10 | 0.80 | 0.05 | 0.66 | 0.12 | 0.95 | 0.23 |
|  | W11 | 0.49 | 0.15 | 0.52 | 0.10 | 0.32 | 0.11 |
|  | W12 | 0.50 | 0.04 | 0.42 | 0.11 | 0.18 | 0.04 |
|  | W13 | 0.44 | 0.07 | 0.42 | 0.04 | 0.42 | 0.02 |
|  | W14 | 0.65 | 0.15 | 0.33 | 0.05 | 0.40 | 0.14 |
|  | W15 | 0.72 | 0.04 | 0.70 | 0.06 | 0.45 | 0.20 |
|  | W16 | 0.66 | 0.03 | 0.24 | 0.05 | 0.38 | 0.11 |
|  | Total | 0.63 | 0.09 | 0.53 | 0.12 | 0.50 | 0.16 |
| Average voltage (V) | W9 | 0.13 | 0.10 | 0.12 | 0.02 | 0.10 | 0.03 |
|  | W10 | 0.15 | 0.03 | 0.15 | 0.04 | 0.14 | 0.05 |
|  | W11 | 0.12 | 0.02 | 0.16 | 0.02 | 0.06 | 0.00 |
|  | W12 | 0.07 | 0.02 | 0.08 | 0.01 | 0.08 | 0.02 |
|  | W13 | 0.23 | 0.03 | 0.19 | 0.07 | 0.12 | 0.01 |
|  | W14 | 0.22 | 0.12 | 0.13 | 0.01 | 0.07 | 0.02 |
|  | W15 | 0.10 | 0.03 | 0.10 | 0.02 | 0.06 | 0.01 |
|  | W16 | 0.09 | 0.03 | 0.07 | 0.02 | 0.06 | 0.00 |
|  | Total | 0.14 | 0.05 | 0.13 | 0.03 | 0.09 | 0.02 |
| Swallowing power (V·s) | W9 | 0.08 | 0.06 | 0.11 | 0.04 | 0.62 | 0.01 |
|  | W10 | 0.11 | 0.02 | 0.09 | 0.02 | 0.11 | 0.02 |
|  | W11 | 0.05 | 0.01 | 0.08 | 0.03 | 0.02 | 0.01 |
|  | W12 | 0.03 | 0.01 | 0.03 | 0.01 | 0.01 | 0.01 |
|  | W13 | 0.10 | 0.01 | 0.08 | 0.03 | 0.05 | 0.01 |
|  | W14 | 0.12 | 0.05 | 0.04 | 0.01 | 0.02 | 0.01 |
|  | W15 | 0.06 | 0.02 | 0.06 | 0.01 | 0.02 | 0.00 |
|  | W16 | 0.05 | 0.01 | 0.02 | 0.01 | 0.02 | 0.01 |
|  | Total | 0.08 | 0.02 | 0.06 | 0.02 | 0.11 | 0.01 |

Data represents mean in three separate measurements

SD: standard deviation
